# Invasive fungal diseases impact on outcome of childhood ALL – an analysis of the international trial AIEOP-BFM ALL 2009

**DOI:** 10.1038/s41375-022-01768-x

**Published:** 2022-12-12

**Authors:** Thomas Lehrnbecher, Andreas H. Groll, Simone Cesaro, Julia Alten, Andishe Attarbaschi, Draga Barbaric, Nicole Bodmer, Valentino Conter, Shai Izraeli, Georg Mann, Anja Möricke, Felix Niggli, Martin Schrappe, Jan Stary, Ester Zapotocka, Martin Zimmermann, Sarah Elitzur

**Affiliations:** 1 Pediatric Hematology and Oncology, Hospital for Children and Adolescents, Johann Wolfgang Goethe-University, Frankfurt, Germany; 2 Infectious Disease Research Program, Department of Pediatric Hematology and Oncology and Center for Bone Marrow Transplantation, University Children’s Hospital Münster, Münster, Germany; 3 Paediatric Haematology Oncology, Department of Mother and Child, Azienda Ospedaliera Universitaria Integrata, Verona, Italy; 4 Pediatrics, University Hospital Schleswig-Holstein, Campus Kiel, Kiel, Germany; 5 St. Anna Kinderspital and Children’s Cancer Research Institute, Vienna, Austria; 6 Sydney Children’s Hospital, Randwick, NSW Australia; 7 University Children’s Hospital Zurich, Zurich, Switzerland; 8 Clinica Pediatrica and Centro Ricerca Tettamanti, Università di Milano-Bicocca, Fondazione MBBM/S.Gerardo Hospital, Monza, Italy; 9 Pediatric Hematology-Oncology, Schneider Children’s Medical Center, Petah Tikva, and Sackler Faculty of Medicine, Tel Aviv University, Tel Aviv, Israel; 10 Czech Working Group for Pediatric Hematology, Prague, Czech Republic; 11 Department of Pediatric Hematology/Oncology, University Hospital Motol, 2nd Faculty of Medicine, Charles University, Prague, Czech Republic; 12 Department of Pediatric Hematology/Oncology, Hannover Medical School, Hannover, Germany

**Keywords:** Epidemiology, Medical research

## Abstract

In children with acute lymphoblastic leukemia (ALL), risk groups for invasive fungal disease (IFD) with need for antifungal prophylaxis are not well characterized, and with the advent of new antifungal compounds, current data on outcome are scarce. Prospectively captured serious adverse event reports of children enrolled in the international, multi-center clinical trial AIEOP-BFM ALL2009 were screened for proven/probable IFD, defined according to the updated EORTC/MSG consensus definitions. In a total of 6136 children (median age 5.2 years), 224 proven/probable IFDs (65 yeast and 159 mold) were reported. By logistic regression, the risk for proven/probable IFDs was significantly increased in children ≥12 years and those with a blast count ≥10% in the bone marrow on day 15 (*P* < 0.0001 each). Proven/probable IFDs had a 6-week and 12-week mortality of 10.7% and 11.2%, respectively. In the multivariate analysis, the hazard ratio for event-free and overall survival was significantly increased for proven/probable IFD, age ≥12 years, and insufficient response to therapy (*P* < 0.001, each). Our data define older children with ALL and those with insufficient treatment-response at high risk for IFD. As we show that IFD is an independent risk factor for event-free and overall survival, these patients may benefit from targeted antifungal prophylaxis.

## Introduction

Acute lymphoblastic leukemia (ALL) is the most common malignancy in childhood and adolescence [1]. Over the last decades, the outcome of pediatric ALL has significantly improved, and in contemporary studies, cure rates exceed 90% [2]. This improvement has been attained by increasing chemotherapy intensity to the limit of tolerance, and therefore, the rates of treatment-related deaths are currently approaching those of relapse-associated mortality.[3] Infections are the most common complications of ALL treatment [4, 5]. For example, in the clinical trial UKALL2003, the 5-year cumulative incidence of infection-related mortality was 2.4%, accounting for 30% of all deaths and more than 60% of treatment-related deaths. Almost 70% of infection-related deaths were caused by bacteria, and 20% by fungi [5].

Risk factors for IFD such as prolonged and profound neutropenia or extended periods of therapy with corticosteroids have been well established [6]. However, as children and adolescents receiving treatment for ALL are a heterogenous group, and the risk for IFD may also depend on additional factors such as age or treatment intensity, it remains unclear which patient subsets could benefit from antifungal prophylaxis. In addition, with the introduction of new diagnostic tools and new classes of antifungal compounds, the outcome of IFD has significantly improved over time [7, 8]. The aim of this analysis was a better characterization of specific risk groups for IFD, and an estimation of outcome in a contemporary setting.

## Patients and methods

### Study population

A total of 6136 children enrolled as study patients in the international, multi-center prospective randomized Phase III clinical trial AIEOP-BFM ALL2009 (EudraCT 2007-004270-43) were included in the analysis. Patients were eligible if they were older than 1 year, younger than 18 years, and were not Ph-positive (BCR/ABL or t(9;22)). The study started June 1, 2010 and ended February 28, 2017 (manuscript submitted). The median time of follow-up was 6 years (1. and 3. quartile, 4.7 and 7.6). Whereas diagnostic procedures (e.g., evaluation of treatment response) and treatment for ALL was prescribed and monitored, supportive care including diagnostics of suspected IFD and antifungal prophylaxis and treatment was at the discretion of the responsible physician. The study was approved by the appropriate national and local review boards, and was conducted in accordance with the Declaration of Helsinki and national laws. Informed consent was obtained from the parents or guardians of each patient included in the study as required by ethical standards and national guidelines.

### Invasive fungal disease (IFD)

Serious adverse events (SAEs) were prospectively captured and reported by the participating institutions. According to study guidelines, IFDs were considered as SAEs, thus, reporting was mandatory. All SAE reports were reviewed and classified by the safety desk of the clinical trial office in Germany (AM/JA), and all SAEs suggestive for IFD were independently reviewed by two experts (TL/AHG). Children were categorized with proven, probable, possible or no IFD according to the revised and updated consensus definitions elaborated by the European Organisation for Research and Treatment of Cancer/Mycoses Study Group (EORTC/MSG) [9]. Hospitals were contacted if relevant data were missing. Conflicting results were harmonized by agreement. All data on patients´ characteristics and diagnosis and treatment of ALL and IFD were retrieved from the AIEOP-BFM database. In case a patient experienced multiple IFDs, only the first episode of an IFD was included in the analysis.

### Statistical analysis

Event-free survival (EFS) was calculated from diagnosis to first event, defined as death during induction therapy, resistance, relapse, death in complete remission, or development of a second malignant neoplasm. Overall survival rates were calculated according to Kaplan–Meier and compared by log-rank test [10, 11]. Cumulative incidence of relapse and treatment-related mortality functions were constructed by the method of *Kalbfleisch and Prentice* and compared with Gray test [12, 13]. Proportional differences between patient groups were analyzed by chi-squared or Fisher’s exact tests, distributions of continuous variables where compared using Wilcoxon two-sample tests. Variables for the uni- and multivariate were chosen based on previous reports [6, 14]. A two tailed *P*-value ≤ 0.05 was considered to be statistically significant. All statistical analyses were performed using the statistical package SAS 9.4 (SAS Institute Inc., SAS Campus Drive, Cary, NC 27513-2414, USA).

## Results

### Incidence and risk factors for IFD

**Fig. 1:**
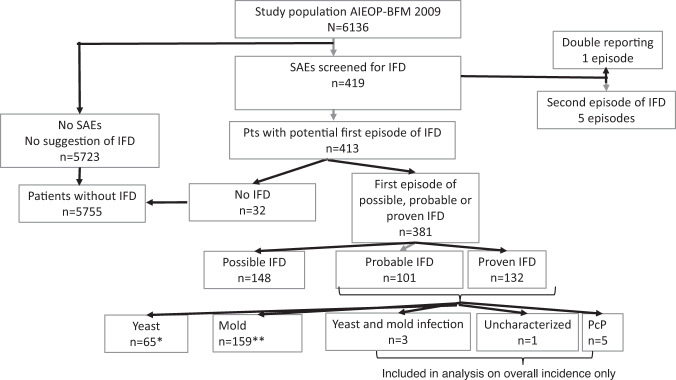
Consort diagram of patients (pts) treated according to the clinical trial AIEOP-BFM 2017 and analyzed for possible, probable and proven invasive fungal disease (IFD). *Yeast infection includes two patients with more than one yeast isolates. **Mold infection includes nine patients with more than one mold isolated. SAE severe adverse event, PcP *Pneumocystis jirovecii* pneumonia.

In 6136 children treated as study patients in the clinical trial AIEOP-BFM ALL2009, a total of 419 SAEs reported on a suspected IFD. Out of these, 381 were identified as the first episode of a possible (*n* = 148), probable (101) or proven (132) IFD (Fig. 1). The 233 episodes of proven/probable IFD consisted of 65 yeast infections (two patients with more than one yeast isolated), 159 mold infections (nine patients with more than one mold isolated), three infections with both yeast and mold isolated, one infection with an uncharacterized fungus and 5 instances of *Pneumocystis jirovecii* pneumonia (PcP) (Fig. 1). The incidence of proven/probable IFD in the study population was 3.8% and that of proven/probable/possible IFD was 6.2%. For further analysis, the nine infections with concurrent yeast and mold infection, uncharacterized fungal infection and PcP, respectively, were excluded, resulting in a total of 6127 patients with 148 possible and 224 proven/probable IFDs (Fig. 1 and Table 1).

**Table 1:** Patient´s characteristics.

		Invasive fungal disease					
	All	No		Possible		Proven/probable	
	*N*	*N*	%	*N*	%	*N*	%
Total	6127	5755	93.9	148	2.4	224	3.7
Male	3536	3332	94.2	83	2.4	121	3.4
Female	2591	2423	93.5	65	2.5	103	4.0
Age							
0–<2 years	421	405	96.2	1	0.2	15	3.6
2–<6 years	3039	2904	95.6	54	1.8	81	2.7
6–<12 years	1579	1495	94.7	41	2.6	43	2.7
12–<15 years	587	525	89.4	25	4.3	37	6.3
15–<18 years	501	426	85.0	27	5.4	48	9.6
Immunophenotype							
Precursor B ALL	5233	4956	94.7	108	2.1	169	3.2
T-ALL	871	778	89.3	39	4.5	54	6.2
Other	16	14	87.5	1	6.3	1	6.3
Down syndrome	151	141	93.4	4	2.6	6	4.0
Blast count in bone marrow on day 15							
<0.1%	2086	1979	94.9	47	2.3	60	2.9
<10%	3028	2880	95.1	59	1.9	89	2.9
≥10%	760	659	86.7	40	5.3	61	8.0
Not evaluable	253	237	93.7	2	0.8	14	5.5
PCR MRD (final)							
Standard	1976	1879	95.1	37	1.9	60	3.0
Intermedium	3059	2893	94.6	76	2.5	90	2.9
medium-SER	383	352	91.9	12	3.1	19	5.0
High	242	215	88.8	8	3.3	19	7.9
Not evaluable	467	416	89.1	15	3.2	36	7.7
Risk group (final)							
Standard risk (SR)	2043	1952	95.5	39	1.9	52	2.5
Intermedium risk (MR)	2681	2553	95.2	49	1.8	79	2.9
High risk (HR)	1403	1250	89.1	60	4.3	93	6.6

The 2591 girls (42.3%) and 3536 boys (57.7%) with a median age (range) of 5.2 years (1-18) were treated in Germany (*n* = 2405), Austria (370), Switzerland (243), Italy (2098), Israel (277), the Czech Republic (407), and Australia (327). Significant differences in the incidence of proven/probable IFD were seen between the countries where the patients were treated (range 1.9% to 7.9%; *P* < 0.0001) (Supplementary Table 1). Gender did not have a significant impact on the risk of proven/probable and possible IFD (*P* = 0.25 and *P* = 0.66, respectively). In the univariate analysis, the risk of proven/probable and possible IFDs significantly differed between the age groups (*P* < 0.0001, each). The highest incidence rates were seen in patients between 15 and 18 years of age (10.1% for proven/probable and 6.0% for possible IFD), whereas the incidence rates were below 4% in patients younger than 12 years (Table). Yeast and mold infections were equally distributed in patients up to the age of 6 years [43 (44.8%) versus 53 (55.2%)], whereas in older patients, mold infections occurred significantly more often than yeast infection: 32 (74.4%) versus 11 (25.6%) in patients between 6 and 12 years of age, and 74 (87.1%) versus 11 (12.9%) in those between 12 and 18 years (*P* < 0.0001). Patients with Down syndrome (*n* = 141) had a similar risk for proven/probable and possible IFD compared to children without Down syndrome (4.3% and 2.8% versus 3.6% and 2.4%; *P* = 0.83 and *P* = 0.85, respectively). In a sub-analysis of 368 patients with data available, the neutrophil count on the day of diagnosis of ALL significantly correlated with the risk for IFD: median number (1. and 3. quartile) of neutrophils per µl in children without IFD, possible IFD and proven/probable IFD 1037 (233; 4026), 6599 (1483; 16,510) and 2163 (1697; 14,660); *P* = 0.027. No significant correlation between neutrophil count and risk of IFD was seen on day 8 after diagnosis of ALL (*P* = 0.63). The neutrophil count at the time of diagnosis also correlated with the initial white blood count (*r* = 0.77; *P* < 0.0001), and children with a white blood count >100,000/mm^3^ at presentation had a significantly higher risk for proven/probable/possible IFD (8.5% versus 5.8%, *P* = 0.018)

**Fig. 2:**
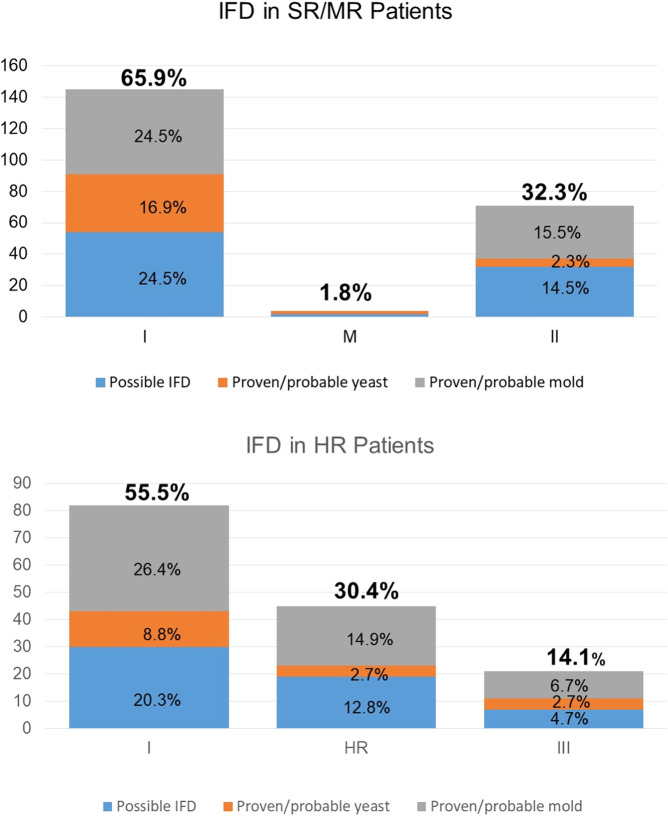
Invasive fungal disease (IFD) in the different treatment phases (protocol I, II, III and HR cycles, respectively) in standard (SR) and intermedium risk (MR) patients (above) and high risk (HR) patients (below). Shown are the absolute number of possible (blue), probable and proven yeast (orange) and probable/proven mold (grey) infections (Y axis). The percentages refer to the total number of IFDs in the respective risk groups (SR/MR and HR).

Univariate analysis of potential risk factors further revealed that insufficient treatment response in the early phase of therapy had a significant impact on the risk for IFD (Supplemental Table 1). Patients with more than 10% leukemic blasts in the bone marrow on day 15 assessed by flow-cytometry had a risk for proven/probable and possible IFD of 8.3% and 5.6%, respectively, compared to a risk for IFD of below 3% when the percentage of leukemic blasts in the bone marrow on day 15 was below 10% (*P* < 0.0001). Similarly, in the univariate analysis, the risk for IFD significantly depended on treatment intensity (*P* < 0.0001). Proven/probable and possible IFD occurred in 6.9% and 4.6% of the high-risk (HR) patients treated more intensively than standard-and intermedium risk (SR/MR) patients who developed proven/probable and possible IFD in less than 3.0%. For all risk groups, the highest risk for IFD was seen in induction and consolidation phase of Protocol I (Fig. 2). Specifically, for the 4718 SR/MR and the 1402 HR patients, a total of 144 and 85 proven/probable/possible IFDs occurred during induction therapy, which represents 65.9% and 55.5% of all proven/probable/possible IFDs diagnosed in SR/MR and HR patients, respectively. Mold infections were seen more often than yeast infections, irrespective of the therapy (Fig. 2).

Logistic regression revealed that the risk for proven/probable IFD was significantly increased in children ≥12 years of age [OR 1.4 (95% CI 1.3; 1.6)] and those with more than 10% blasts in the bone marrow on day 15 assessed by flow-cytometry [OR 2.3 (95% CI 1.7; 3.2)] (*P* < 0.0001 each) (Supplemental Table 2).

### Impact of IFD on duration of intensive chemotherapy

Compared to patients without IFD, patients with proven/probable and patients with possible IFD had a significantly longer duration of intensive chemotherapy (measured from start of intensive chemotherapy until start of maintenance therapy). The median duration (years; 1. and 3. quartile) of intensive chemotherapy was 0.69 (0.65; 0.73), 0.69 (0.65; 0.74) and 1.17 (1.09; 1.26) in SR, MR, and HR patients without IFD, 0.74 (0.69; 0.77), 0.72 (0.68; 0.82), and 1.26 (1.15; 1.35) in SR, MR and HR patients with possible IFD versus 0.77 (0.72; 0.86), 0.76 (0.71; 0.88) and 1.21 (1.14; 1.37) in SR, MR and HR patients with proven/probable IFD, respectively. The differences between patients with and without IFD were significant for each of the risk groups (SR and MR *P* < 0.0001 each, HR *P* = 0.0004).

### Outcome

**Fig. 3:**
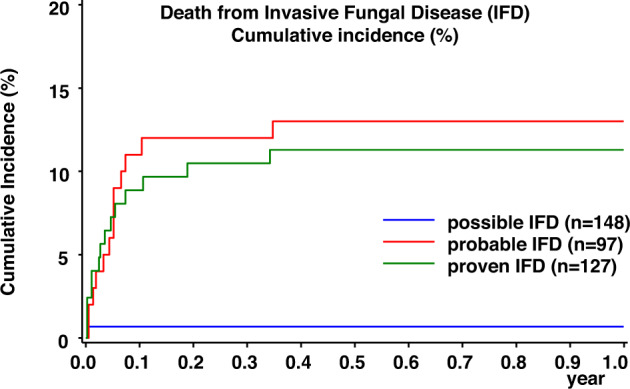
Cumulative incidence of death from possible (blue), probable (red) and proven (green) invasive fungal disease (IFD) in children treated for acute lymphoblastic leukemia. The *X*-axis represents the time period of one year after diagnosis of IFD.

A total of 24 and 25 patients suffering from proven/probable IFD did not survive the infection after 6 and 12 weeks, accounting for a 6-week and 12-week mortality rate of 10.7% and 11.2%, respectively (Fig. 3). After 6 weeks, 4 with children with yeast infection and 20 children with proven/probable mold infection did not survive the infection (after 12 weeks, 4 and 21 patients, respectively). Overall mortality 1 year after IFD diagnosis was 12.1% (27 out of 224 patients), and was 6.1% for patients with yeast infection (4 out of 65 patients) and 14.5% (23 out of 159) for patients with mold infection (difference not significant; *P* = 0.08). Mortality following IFD of all study patients was 0.44%.

In the multivariate analysis, the hazard ratio [95% confidence interval (CI)] for 5-year EFS was significant for proven/probable IFD (1.86, 1.42-2.45), age ≥12 years (1.14, 1.07–1.21), insufficient response to therapy [blast count on day 15 measured by flow- cytometry ≥10% (1.52, 1.26–1.82) or minimal residual disease measured by PCR on day 33 (2.08, 1.76–2.45); *P* < 0.001 each] (Supplemental Table 3). Similarly, the hazard ratio (95% CI) for 5-year OS was significant for proven/probable IFD (2.51, 1.79–3.51), age ≥12 years (1.32, 1.21–2.15), insufficient response to therapy [blast count on day 15 (1.74, 1.35–2.24) and minimal residual disease on day 33 (2.61, 2.04–3.34); *P* < 0.001 each]. The hazard ratio for both 5-year EFS and 5-year OS was significantly lower for patients with ETV6/RUNX1 0.62, 0.50–0.77; P < 0.001 and 0.52, 0.35–0.78; *P* = 0.002, respectively.

## Discussion

In the prospective, randomized multi-international clinical trial AIEOP-BFM ALL2009 enrolling 6136 children with ALL, the reported overall incidence of proven/probable IFD and proven/probable/possible IFD was 3.8% and 6.2%, respectively, but was significantly higher in various subgroups, e.g., in older children and adolescents or in patients with insufficient early treatment response. The reported incidence rates of IFD in children with ALL vary widely across previous studies with rates reaching from 3.8% to up to 24% [15–17]. These differences can be explained, at least in part, by the fact that the studies, all of which included considerably fewer patients than the present clinical trial, varied by the use of diagnostic tools and definition of IFD [15, 18–20]. In addition, local epidemiology has an important impact on both the risk for IFD and the predominant pathogen isolated, as also supported by the data of our multi-international trial [8, 15, 17, 18, 20, 21]. Our analysis demonstrated by both univariate and multivariate analysis that older age is associated with a higher risk for IFD. Whereas previous data on the correlation of age and risk for IFD were conflicting in children with ALL and IFD [15, 18], this correlation has been found in children with AML or in those after allogeneic hematopoietic stem cell transplantation [6]. In line with our results, other reports demonstrated that children receiving higher intensity of treatment, such as HR patients, are at a higher risk for IFD [8, 15]. However, it is important to note that in the clinical trial AIEOP-BFM ALL2009, HR patients received the same treatment as SR and MR patients during induction therapy, the time period in which the majority of IFDs occurred. We further observed that insufficient treatment response during induction therapy is an important criterion for risk stratification for IFD, which we describe here for the first time as an independent risk factor.

Unexpectedly, we found in a subgroup of patients a significant correlation between the number of neutrophils at the time of diagnosis of ALL and the risk for IFD. This is in contrast to children with acute myeloid leukemia (AML), in which neutropenia at the start of chemotherapy was independently associated with IFD [22]. Our surprising result might be explained by the facts that the neutrophil count at the time of diagnosis correlated with the initial white blood count, and that patients with a white blood count >100,000/mm^3^ at presentation, which has been described as an unfavorable prognostic variable, had a significantly higher risk for proven/probable/possible IFD [23, 24]. Notably, the oxidative burst activity is significantly reduced in children with ALL at the time of diagnosis and normalizes after consolidation therapy, which indicates that ALL blasts hamper the antifungal host immune response [25]. In addition, the release of neutrophil extracellular traps (NETs), which play an important role in the defense against various fungi such as *Aspergillus* or *Candida*, is significantly impaired in children with ALL at the time of diagnosis, and full restoration of neutrophil function is achieved only after successful completion of leukemia treatment [26, 27]. We did not analyze whether the time of profound neutropenia correlated with the risk of IFD, as we have not captured a close monitoring of neutrophil counts and as this risk factor has well been described by a number of pediatric and adult studies [6, 28]. In addition, it is important to note that in the AIEOP-BFM ALL2009 trial, corticosteroids have been administered for the first 6 weeks to all patients, which have a pleiotropic paralyzing effect on neutrophils and macrophages [29].

In an early report on invasive aspergillosis in 66 children with cancer published in the 1990ies, mortality after 1 month was 48%, and was 75% after 2 months [7]. Fortunately, with the availability of new amphotericin B formulations and new antifungal compounds of different classes, such as broad-spectrum triazoles including voriconazole and posaconazole or echinocandins including micafungin and caspofungin, mortality rates appear to have significantly decreased. The 6- and 12-week mortality rates of 10.7% and 11.2% in our study were comparable with other recent results [8], but notably, mortality rates of studies still vary widely and range from 0% to up to 44% [19, 20]. Corroborating other analyses [15, 18], our results demonstrate that IFD delays chemotherapy. As a result, treatment intensity over time was significantly reduced by IFD, which was seen across all ALL risk groups. This observation might explain, at least in part, that proven/probable IFD is an independent and significant factor with impact not only on overall survival, but also on EFS, which has not been reported before. As IFD impairs the timely administration of chemotherapy, the differentiation between crude and therapy-associated mortality is even more challenging [30]. However, it is important to note that small studies suggest that new agents such as blinatumomab may be used as bridging therapy in pediatric ALL patients suffering from IFD [31, 32].

The strength of our study is the fact that the analysis is based on the data of a contemporary multi-centered international randomized controlled clinical trial. We included the largest dataset on IFD in pediatric ALL to date, and the data quality is extremely high due to the fact that SAEs were prospectively collected and monitored in a systematic way. In addition, all potentially relevant SAEs were independently evaluated by two experts, based on the revised and updated definitions of IFD from the EORTC/MSG consensus group, which added the widely used *Aspergillus* PCR as mycological criterion for probable invasive aspergillosis [9]. However, we recognize that our analysis also has limitations. Most importantly, we do not have data on the use of antifungal prophylaxis, which may have impacted on the incidence for IFD. On the other hand, in contrast to AML [33, 34], data on a significant effect of antifungal prophylaxis in patients with ALL is lacking for both children and adults [17, 35], and widely used prophylactic options such as the intermittent administration of liposomal amphotericin B or micafungin have not demonstrated significant antifungal efficacy in any randomized trial [36, 37]. Another limitation of our study is the fact that the use of important diagnostic tools such as pulmonary CT scan or galactomannan testing was at the discretion of the institution where the patient was treated, which is an inherent problem of all analyses of uncontrolled data.

## Conclusion

In conclusion, our data show that the overall incidence rate of IFD in pediatric ALL is relatively low, but independent risk factors such as older age and treatment-response can define high risk groups for IFD. Based on this characterization of risk groups, randomized trials on antifungal prophylaxis can be designed, which may ultimately improve the overall outcome of ALL, as our data demonstrate that proven/probable IFD is an independent risk factor for both event-free and overall survival of children with ALL.

## Supplementary information

Supplemental Tables

## Data Availability

Under the permission that national data protection requirements are fully met, access to individual data may be made available upon reasonable request to the authors.

## References

[1] Smith MA, Altekruse SF, Adamson PC, Reaman GH, Seibel NL (2014). Declining childhood and adolescent cancer mortality. Cancer.

[2] Hough R, Vora A (2017). Crisis management in the treatment of childhood acute lymphoblastic leukemia: putting right what can go wrong (emergency complications of disease and treatment). Hematol Am Soc Hematol Educ Program.

[3] Essig S, Li Q, Chen Y, Hitzler J, Leisenring W, Greenberg M (2014). Risk of late effects of treatment in children newly diagnosed with standard-risk acute lymphoblastic leukaemia: a report from the Childhood Cancer Survivor Study cohort. Lancet Oncol.

[4] Lund B, Asberg A, Heyman M, Kanerva J, Harila-Saari A, Hasle H (2011). Risk factors for treatment related mortality in childhood acute lymphoblastic leukaemia. Pediatr Blood Cancer.

[5] O’Connor D, Bate J, Wade R, Clack R, Dhir S, Hough R (2014). Infection-related mortality in children with acute lymphoblastic leukemia: an analysis of infectious deaths on UKALL2003. Blood.

[6] Fisher BT, Robinson PD, Lehrnbecher T, Steinbach WJ, Zaoutis TE, Phillips B (2018). Risk Factors for Invasive Fungal Disease in Pediatric Cancer and Hematopoietic Stem Cell Transplantation: A Systematic Review. J Pediatr Infect Dis Soc.

[7] Abbasi S, Shenep JL, Hughes WT, Flynn PM (1999). Aspergillosis in children with cancer: A 34-year experience. Clin Infect Dis.

[8] Wang SS, Kotecha RS, Bernard A, Blyth CC, McMullan BJ, Cann MP (2019). Invasive fungal infections in children with acute lymphoblastic leukaemia: Results from four Australian centres, 2003-2013. Pediatr Blood Cancer.

[9] Donnelly JP, Chen SC, Kauffman CA, Steinbach WJ, Baddley JW, Verweij PE (2020). Revision and update of the consensus definitions of invasive fungal disease from the European organization for research and treatment of cancer and the mycoses study group education and research consortium. Clin Infect Dis.

[10] Kaplan EL, Meier P (1958). Nonparametric estimation from incomplete observations. J Am Stat Assoc.

[11] Mantel N (1966). Evaluation of survival data and two new rank order statistics arising in its consideration. Cancer Chemother Rep..

[12] Kalbfleisch JD, Prentice RL, The statistical analysis of failure time data. New York: John Wiley; 1980. p. 163–88.

[13] Gray RJ (1988). A class of K-sample tests for comparing the cumulative incidence of a competing risk. Ann Stat.

[14] Schmidt MP, Colita A, Ivanov AV, Coriu D, Miron IC (2021). Outcomes of patients with Down syndrome and acute leukemia: A retrospective observational study. Med (Baltim).

[15] O’Reilly MA, Govender D, Kirkwood AA, Vora A, Samarasinghe S, Khwaja A (2019). The incidence of invasive fungal infections in children, adolescents and young adults with acute lymphoblastic leukaemia/lymphoma treated with the UKALL2011 protocol: a multicentre retrospective study. Br J Haematol.

[16] Sahbudak Bal Z, Yilmaz Karapinar D, Karadas N, Sen S, Onder Sivis Z, Akinci AB (2015). Proven and probable invasive fungal infections in children with acute lymphoblastic leukaemia: results from an university hospital, 2005-2013. Mycoses.

[17] Olivier-Gougenheim L, Rama N, Dupont D, Saultier P, Leverger G, AbouChahla W (2021). Invasive Fungal Infections in Immunocompromised Children: Novel Insight Following a National Study. J Pediatr.

[18] Jain S, Kapoor G (2015). Invasive aspergillosis in children with acute leukemia at a resource-limited oncology center. J Pediatr Hematol Oncol.

[19] Inaba H, Pei D, Wolf J, Howard SC, Hayden RT, Go M (2017). Infection-related complications during treatment for childhood acute lymphoblastic leukemia. Ann Oncol.

[20] Das A, Oberoi S, Trehan A, Chakrabarti A, Bansal D, Saxena AK (2018). Invasive Fungal Disease in Pediatric Acute Leukemia in the Nontransplant Setting: 8 Years’ Experience From a Tertiary Care Center in North India. J Pediatr Hematol Oncol.

[21] Zawitkowska J, Drabko K, Szmydki-Baran A, Zaucha-Prazmo A, Lejman M, Czyzewski K (2019). Infectious profile in children with ALL during chemotherapy: A report of study group for infections. J Infect Chemother.

[22] Johnston DL, Lewis V, Yanofsky R, Gillmeister B, Ethier MC, Mitchell D (2013). Invasive fungal infections in paediatric acute myeloid leukaemia. Mycoses.

[23] Teachey DT, Hunger SP (2013). Predicting relapse risk in childhood acute lymphoblastic leukaemia. Br J Haematol.

[24] Schultz KR, Pullen DJ, Sather HN, Shuster JJ, Devidas M, Borowitz MJ (2007). Risk- and response-based classification of childhood B-precursor acute lymphoblastic leukemia: a combined analysis of prognostic markers from the Pediatric Oncology Group (POG) and Children’s Cancer Group (CCG). Blood.

[25] Tanaka F, Goto H, Yokosuka T, Yanagimachi M, Kajiwara R, Naruto T (2009). Suppressed neutrophil function in children with acute lymphoblastic leukemia. Int J Hematol.

[26] Ostafin M, Ciepiela O, Pruchniak M, Wachowska M, Ulinska E, Mrowka P, et al. Dynamic Changes in the Ability to Release Neutrophil ExtraCellular Traps in the Course of Childhood Acute Leukemias. Int J Mol Sci. 2021;22. 10.3390/ijms22020821 PMC7829911 33467555

[27] Urban CF, Nett JE (2019). Neutrophil extracellular traps in fungal infection. Semin Cell Dev Biol.

[28] Taplitz RA, Kennedy EB, Bow EJ, Crews J, Gleason C, Hawley DK (2018). Antimicrobial Prophylaxis for Adult Patients With Cancer-Related Immunosuppression: ASCO and IDSA Clinical Practice Guideline Update. J Clin Oncol.

[29] Lehrnbecher T, Foster C, Vazquez N, Mackall CL, Chanock SJ (1997). Therapy-induced alterations in host defense in children receiving chemotherapy. J Ped Hematol Oncol.

[30] Alexander S, Pole JD, Gibson P, Lee M, Hesser T, Chi SN (2015). Classification of treatment-related mortality in children with cancer: a systematic assessment. Lancet Oncol.

[31] Yeoh DK, Blyth CC, Kotecha RS (2022). Blinatumomab as bridging therapy in paediatric B-cell acute lymphoblastic leukaemia complicated by invasive fungal disease. Br J Haematol.

[32] Elitzur S, Arad-Cohen N, Barzilai-Birenboim S, Ben-Harush M, Bielorai B, Elhasid R (2019). Blinatumomab as a bridge to further therapy in cases of overwhelming toxicity in pediatric B-cell precursor acute lymphoblastic leukemia: Report from the Israeli Study Group of Childhood Leukemia. Pediatr Blood Cancer.

[33] Fisher BT, Zaoutis T, Dvorak CC, Nieder M, Zerr D, Wingard JR (2019). Effect of caspofungin vs fluconazole prophylaxis on invasive fungal disease among children and young adults with acute myeloid leukemia: a randomized clinical trial. JAMA.

[34] Cornely OA, Maertens J, Winston DJ, Perfect J, Ullmann AJ, Walsh TJ (2007). Posaconazole vs. fluconazole or itraconazole prophylaxis in patients with neutropenia. N. Engl J Med.

[35] Cornely OA, Leguay T, Maertens J, Vehreschild M, Anagnostopoulos A, Castagnola C (2017). Randomized comparison of liposomal amphotericin B versus placebo to prevent invasive mycoses in acute lymphoblastic leukaemia. J Antimicrob Chemother.

[36] Bochennek K, Tramsen L, Schedler N, Becker M, Klingebiel T, Groll AH (2011). Liposomal amphotericin B twice weekly as antifungal prophylaxis in pediatric high risk patients. Clin Micro Infect.

[37] Bochennek K, Balan A, Muller-Scholden L, Becker M, Farowski F, Muller C (2015). Micafungin twice weekly as antifungal prophylaxis in paediatric patients at high risk for invasive fungal disease. J Antimicrob Chemother.

