# Supplementary material for: Invasive fungal diseases impact on outcome of childhood ALL – an analysis of the international trial AIEOP-BFM ALL 2009
Source: Leukemia. 2022 Dec 12;37(1):72–8. doi: 10.1038/s41375-022-01768-x (PMC9883161; doi:10.1038/s41375-022-01768-x)
Supplement: Supplementary file 1 — Supplemental Tables [file 41375_2022_1768_MOESM1_ESM.docx]

**Supplemental Table 1:** Univariate analysis of potential factors influencing the risk for invasive fungal disease

|  |  |  | **Univariate analysis** |
| --- | --- | --- | --- |
|  | **No IFD [n (%)]** | **Proven/probable IFD [n (%)]** | **P value** |
| **Country** |  |  |  |
| Italy | 1985 (97.1) | 60 (2.9) | <0.0001 |
| Austria | 360 (98.1) | 7 (1.9) |  |
| Switzerland | 231 (96.7) | 8 (3.3) |  |
| Germany | 2257 (96.5) | 81 (3.5) |  |
| Czech Republic | 375 (94.0) | 24 (6.0) |  |
| Australia | 292 (92.1) | 25 (7.9) |  |
| Israel | 255 (93.1) | 19 (6.9) |  |
| **Sex** |  |  | 0.25 |
| male | 3332 (96.5) | 121 (3.5) |  |
| female | 2423 (95.9) | 103 (4.1) |  |
| **Age** |  |  | <0.0001 |
| 0-<2 years | 405 (96.4) | 15 (3.6) |  |
| 2-<6 years | 2904 (97.3) | 81 (2.7) |  |
| 6-<12 years | 1495 (97.2) | 43 (2.8) |  |
| 12-<15 years | 525 (93.4) | 37 (6.6) |  |
| 15-<18 years | 426 (89.9) | 48 (10.1) |  |
| **Down Syndrome** |  |  |  |
| no | 5614 (96.3) | 218 (3.7) | 0.83 |
| yes | 141 (95.9) | 6 (4.1) |  |
| **Immunophenotype*** |  |  |  |
| Precursor B All | 4956 (96.7) | 169 (3.3) | <0.0001 |
| T-ALL | 778 (93.5) | 54 (6.5) |  |
| **Blast count in bone marrow on day 15 by flow-cytometry** |  |  |  |
| <0.1% | 1979 (97.1) | 61 (2.9) | <0.0001 |
| <10% | 2880 (97.0) | 89 (3.0) |  |
| ≥10% | 659 (91.5) | 61 (8.5) |  |
| **PCR MRD (final)*** |  |  |  |
| Standard | 1879 (96.9) | 60 (3.1) | <0.0001 |
| Intermedium | 2893 (97.0) | 90 (3.0 |  |
| medium-SER | 352 (94.9) | 19 (5.1) |  |
| High | 215 (92.0) | 19 (8.1) |  |
| **Risk group (final)** |  |  |  |
| Standard risk (SR) | 1952 (97.4) | 52 (2.6) | <0.0001 |
| Intermedium risk (MR) | 2553 (97.0) | 79 (3.0) |  |
| High risk (HR) | 1250 (93.1) | 93 (6.9) |  |
|  |  |  |  |
| IFD invasive fungal disease  *only evaluable data included | | |  |

**Supplemental Table 2:** Multivariate analysis of potential factors influencing the risk for invasive fungal disease

|  |  |  | **Multivariate analysis** | | |
| --- | --- | --- | --- | --- | --- |
| **#** | **No IFD (n (%)** | **Proven/probable IFD (n (%))** | **P value** | **OR** | **95% CI** |
| **Sex** |  |  |  |  |  |
| male | 3332 (96.5) | 121 (3.5) | 0.09 | 1.3 | 0.96-1.7 |
| female | 2423 (95.9) | 103 (4.1) |  |  |  |
| **Age** |  |  |  |  |  |
| 0-<12 years | 4804 (97.2) | 139 (2.9) | <0.0001 | 2.8 | 2.1-3.8 |
| 12-<18 years | 951 (91.8) | 85 (8.2) |  |  |  |
| **Down Syndrome** |  |  |  |  |  |
| no | 5614 (96.3) | 218 (3.7) | 0.88 | 0.93 | 0.37-2.3 |
| yes | 141 (95.9) | 6 (4.1) |  |  |  |
| **Immunophenotype*** |  |  |  |  |  |
| Precursor B ALL | 4956 (96.7) | 169 (3.3) | 0.12 | 1.3 | 0.9-1.9 |
| T-ALL | 778 (93.5) | 54 (6.5) |  |  |  |
| **Blast count in bone marrow on day 15 by FCM** |  |  |  |  |  |
| <10.% | 4849 (97.0) | 150 (3.0) | <0.0001 | 2.3 | 1.7-3.2 |
| ≥10% | 659 (91.5) | 61 (8.5) |  |  |  |

OR Odds ratio; CI confidence interval; IFD invasive fungal disease

*only evaluable data included

# first row standard

**Supplemental Table 3:** Multivariate analysis of factors influencing event-free survival and overall survival

|  | **Event-free survival** | | |  | **Overall survival** | | |
| --- | --- | --- | --- | --- | --- | --- | --- |
| **#** | **P** | **Hazard ratio** | **95% CI** |  | **P** | **Hazard ratio** | **95% CI** |
| **Age** |  |  |  |  |  |  |  |
| 0-<12 years | <0.001 | 1.34 | 1.14-1.57 |  | <0.001 | 1.73 | 1.38-2.16 |
| 12-<18 years |  |  |  |  |  |  |  |
| **Proven/probable IFD** |  |  |  |  |  |  |  |
| no | <0.001 | 1.88 | 1.43-2.47 |  | <0.001 | 2.58 | 1.85-3.61 |
| yes |  |  |  |  |  |  |  |
| **Immunophenotype*** |  |  |  |  |  |  |  |
| Precursor B All | 0.026 | 0.80 | 0.66-0.97 |  | 0.07 | 1.25 | 0.98-1.60 |
| T-ALL |  |  |  |  |  |  |  |
| **Genetic aberration** |  |  |  |  |  |  |  |
| others | <0.001 | 0.62 | 0.50-0.77 |  | <0.001 | 0.50 | 0.34-0.75 |
| ETV6/RUNX1 |  |  |  |  |  |  |  |
| **Blast count in bone marrow on day 15** |  |  |  |  |  |  |  |
| <10.% | <0.001 | 1.52 | 1.26-1.83 |  | <0.001 | 1.76 | 1.36-2.27 |
| ≥10% |  |  |  |  |  |  |  |
| **PCR MRD (final)*** |  |  |  |  |  |  |  |
| SR/MR | <0.001 | 2.09 | 1.77-2.47 |  | <0.001 | 2.668 | 2.08-3.4 |
| High |  |  |  |  |  |  |  |
|  |  |  |  |  |  |  |  |
| CI confidence interval; IFD invasive fungal disease  *only evaluable data included  # first row standard | | |  |  |  |  |  |
